# Supplementary material for: Automation protocol for high-efficiency and high-quality genomic DNA extraction from Saccharomyces cerevisiae
Source: PLoS One. 2023 Oct 17;18(10):e0292401. doi: 10.1371/journal.pone.0292401 (PMC10581484; doi:10.1371/journal.pone.0292401)
Supplement: S1 File — (PDF) [file pone.0292401.s001.pdf]

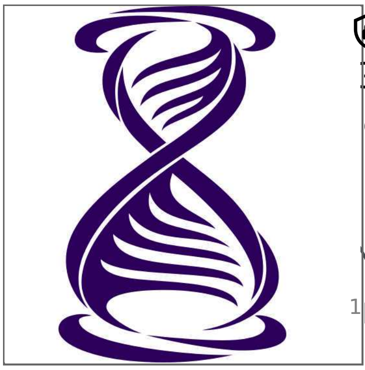

CY2KXYCW

# Automation Protocol for High-Efficiency and High-Quality Genomic DNA Extraction from Saccharomyces Cerevisiae V. (cy2kxycw)

Nina Alperovich[1], David Ross[1], Benjamin M Scott[2]

[1]NIST; [2]Global Institute for Food Security

David Ross

**Protocol Info:** Nina Alperovich, David Ross, Benjamin M Scott . Automation Protocol for High-Efficiency and High-Quality Genomic DNA Extraction from Saccharomyces Cerevisiae. **protocols.io** https://protocols.io/view/automation-protocol-for-high-efficiency-and-high-q-cy2kxycwVersion created by David Ross

**Created:** Aug 22, 2023

**Last Modified:** Aug 22, 2023

**PROTOCOL integer ID:** 86828

**Keywords:** laboratory automation, synthetic biology, Saccharomyces Cerevisiae, DNA extraction

## DISCLAIMER

Certain commercial equipment, instruments, or materials are identified to adequately specify experimental procedures. Such identification neither implies recommendation nor endorsement by the National Institute of Standards and Technology nor that the equipment, instruments, or materials identified are necessarily the best for the purpose.

## ABSTRACT

Here, we describe a protocol for automated extraction of genomic DNA (gDNA) from yeast liquid culture and colonies. The protocol uses a Hamilton NGS-STAR automated liquid handler equipped with thermal-regulated microplate shakers. We have tested this automated protocol with 1.5 mL aliquots of liquid culture ($OD_{600}$= 1) to demonstrate that it can provide high-quality gDNA at high efficiency. The protocol can also be implemented with manual pipetting, and/or to extract gDNA from larger volumes of culture by proportionally increasing the volumes of reagents and using an appropriate magnet separation rack.

MATERIALS

**Reagents:**
• PBS, pH 7.4 (Invitrogen, cat. no. AM9625)
• SDS, 20% (Quality Biological, cat. no. 351-066-101)
• EDTA, 0.5 mol/L, pH 8.0 (Ambion, cat. no.AM9262)
• TRIS-HCl, 1 mol/L, pH 8.0 (Invitrogen, cat.no. 15568)
• 1x TE Buffer, pH 8.0 (ThermoFisher Scientific, cat. no. 12090015)
• Polyethylene glycol (PEG-8000, Sigma, cat. no. 89510)
• Sodium Chloride, 5 mol/L (Invitrogen, cat.no. AM9760G)
• Zymolyase 20T (US Biological, Cas. No. 37340-57-1)
• 2-Mercaptoethanol (ThermoFisher Scientific, Cas. no. 60-24-2)
• RNase A/T1 Mix (ThermoFisher Scientific, cat. no. EN0551)
• Speed-Bead Magnetic Carboxylate Modified Particle (Cytiva, cat. no. 65152105050250)
• TWEEN-20 (Life technologies, cat. no. 003005)
• Ethanol, 200 proof (100%) (Fisher Scientific, cat. # 2716TR)
• Nuclease-Free Water (ThermoFisher Scientific, cat. no. AM9938)
• Elution Buffer: 10 mmol/L TRIS-HCl, pH 8.0 (Invitrogen, cat.no. 15568)
• Optional (for step 43): Proteinase K, recombinant, PCR grade, 20mg/mL, (ThermoFisher Scientific cat. no. EO0491)

**Additional labware:**
• Spin Plate: 96-well deep-well plate (Eppendorf, cat. no. 951033405)
• Reaction Plate: 96-well midi plate (Abgene, AB-0765)
• Elution plate: Low-binding 96-well (Eppendorf, cat. no. 30603303)
• Universal Lid: (Agilent, cat. no. 202497-100)
• 12-Tube Magnetic Separation Rack (New England Biolabs, cat.no. S1509)
• Aluminum Foil Lids (Beckman Coulter, cat. no. 538619)
• Rubber Roller (Beckman Coulter, cat. no. 538619)
• 50 mL Conical Tubes (FALCON, cat.no. 352098)
• Microcentrifuge Tubes, 1.7 mL, RNase/DNase free (Costar, cat. no. 3207/3620)

**Equipment:**
• Automated liquid handler (Hamilton Robotics, NGS-STAR).
• Centrifuge for 96-well plates.

## Preparation of Zymolyase Stock

1 Prepare stock of Zymolyase 20T by dissolving to a concentration of 1 U/µL in 1x PBS.

2 Divide Zymolyase solution into 800 µL aliquots and store at -20 °C until use.

## Preparation of 2-Mercaptoethanol Stock

3 Prepare 0.001 % stock of 2-Mercaptoethanol by diluting 1000-fold into 1x PBS.

4 Store at 4 °C until use.

## Preparation of Digestion Master Mix

5 Add 22.72 mL 1x PBS

6 Add one aliquot (800 µL) of Zymolyase Stock, 1 U/µL

7 Add 160 µL 0.001 % 2-Mercaptoethanol

8 Add 320 µL RNase A/T1 mix

9 Prepare fresh before each use

## Preparation of 4x Lysate Buffer

10 Add 30 mL Nuclease-Free Water.

11 Add 60 mL 20% SDS

12 Add 4 mL 0.5 mol/L EDTA.

13 Add 6 mL 1 mol/L TRIS-HCL, pH 8.0

14 Store at 4 °C until use.

## Preparation of Magnetic Beads Stock

15 Vortex Speed-Bead Magnetic beads to resuspend

16 Divide Speed-Bead Magnetic beads into 1 mL aliquots, each in in a 1.7 mL Microcentrifuge Tube.

17 Place micro-centrifuge tubes containing magnetic beads solutions on a 12-Tube Magnetic Separation Rack until beads are drawn to the magnet and solutions are clear.

18 Remove supernatant.

19 Add 1 mL 1x TE Buffer to each micro-centrifuge tube containing beads, remove from magnetic stand, and mix by vortex.

20 Repeated steps 17-19 two additional times.

21 Store bead stock in the dark at 4 °C. Bring bead stock to room temperature and mix well by vortexing before use.

## Preparation of Magnetic Bead/PEG-NaCl Binding Buffer

22 Add 9 g PEG-8000 to a new 50 mL Conical Tube.

23 Add 10 mL 5 mol/L Sodium Chloride.

24 Add 500 µL 1 mol/L Tris-HCl, pH8.

25 Add 500 µL 0.5 mol/L EDTA.

26 Fill Conical Tube to the 40 mL mark with Nuclease-Free Water, then vortex to mix every 1-2 minutes until PEG goes into solution.

27 Add 27.5 µL Tween-20 and mix gently by inverting 4-6 times.

28 Vortex Magnetic Beads Stock (from previous section) to mix thoroughly.

29 Add Magnetic Bead Stock:

29.1 For extraction of gDNA from liquid culture, add 3 mL Magnetic Beads Stock to the 50 mL Conical Tube containing the PEG/NaCl solution.

29.2 For extraction of gDNA from yeast colonies, add 1 mL Magnetic Beads Stock to the 50 mL Conical Tube containing the PEG/NaCl solution.

30 Fill Conical Tube to the 50 mL mark with Nuclease-Free Water, and gently mix until solution is a uniform brown color.

31 Wrap in foil (or place in dark container) and store at 4 °C, mix well by vortexing before use.

## Preparation of yeast liquid culture for extraction

32 Aliquot 1.5 mL (OD600=1) in 1-3 columns of 96-well Spin Plate using automated multichannel pipettes.

33 Centrifuge at 3700 g for 10 min at 10 °C.

34 Remove supernatant from each well using automated liquid handler.

34.1 Use modified labware definition or other constraints on automated pipetting to set a minimum distance of 2.5 mm between the bottom of each well and the tip of each pipette.

34.2 The resulting volume of the remaining cell pellet and residual liquid should be approximately 50 µL.

35 Seal Spin Plate using Aluminum Foil Lid and Rubber Roller.

36 Store at -20 °C.

37 Defrost at 4 °C for 30-60 minutes before continuing with extraction.

## Preparation of yeast colonies for extraction

38 Pick up colonies from agar plate and resuspend each in 50 µL of PBS in 1-3 columns of 96-well Spin Plate.

39 Proceed with DNA extraction from resuspended colonies as described below.

## Yeast genomic DNA extraction. The following steps are imple...

40 Pipette 315 µL of Digestion Master Mix into each well containing a cell pellet or resuspended

colony; mix 30 times by repeated aspiration and dispense.

41 Transfer 315 µL from each well containing resuspended cell sample to a well in a new Reaction Plate located on a thermal-regulated microplate shaker.

42 Incubate at 37 °C for 25 minutes while shaking at 400 rpm.

43 Optional (if protein contamination is problematic for down-stream application): Pipette 10 uL of Proteinase K solution to each well.

44 Pipette 100 µL of 4x Lysate Buffer to each well and mix 3 times by repeated aspiration and dispense.

45 Optional (if Proteinase K was added in step 43): Incubate at 50°C for 25 minutes.

46 Incubate at 75°C for 10 minutes.

47 Move the Reaction Plate to a microplate shaker at room temperature.

48 Pipette 200 µL Magnetic Bead/PEG-NaCl Binding Buffer into each well.

48.1 Mix Magnetic Bead/PEG-NaCl Binding Buffer well by repeated aspiration and dispense before each transfer.

48.2 After dispensing beads into each well, mix beads with cell lysate with one aspiration and dispense.

48.3 During this step, the ratio of Magnetic Bead/PEG-NaCl Binding Buffer to the lysed sample volume is 0.48 (200 µL : 415 µL). Consequently, because of the relatively low PEG concentration in the mixture, only high molecular weight chromosomal DNA will bind to the beads.

49 Incubate at room temperature for 7 minutes while shaking at 300 rpm.

50 Move the Reaction Plate to a 96-well magnet base to pull the magnetic beads out of suspension

51 Wait for 5 minutes.

52 Remove and discard supernatant from each sample (approximately 615 µL).

53 Add 600 µL 80 % Ethanol to each well.

54 Wait for 1 minute.

55 Remove and discard the ethanol supernatant.

56 Add 300 µL 80 % Ethanol to each well.

57 Wait for 1 minute.

58 Remove and discard the ethanol supernatant.

58.1 After removing most of the supernatant with large-volume tips, use smaller-volume tips (e.g., 50 µL) to remove any residual supernatant from the bottom of each well.

59 Allow magnetic bead pellets to dry for 5 minutes at room temperature.

60 Move the Reaction Plate from magnet base to an automated thermal-regulated microplate shaker with temperature set to 60 °C.

61 Add 100 µL Nuclease-Free Water to each well (note: can also use EB)

62 Resuspend beads by repeated aspiration and dispense (12 times).

63 Incubate at 60 °C for 10 s while shaking at 1000 rpm.

64 Incubate at 60 °C for 7 minutes while shaking at 400 rpm.

65 Move the Reaction Plate back to the magnet base.

66 Wait for 5 minutes.

67 Transfer the supernatant from each well to a new, clean well in the same plate (still on the magnet base).

68 Wait for 5 minutes.

69 Transfer the supernatant with extracted DNA from each well to a well in the Elution plate.

70 Cover the Elution plate containing the extracted DNA samples with a Universal Lid.
