# Supplementary material for: Automation protocol for high-efficiency and high-quality genomic DNA extraction from Saccharomyces cerevisiae
Source: PLoS One. 2023 Oct 17;18(10):e0292401. doi: 10.1371/journal.pone.0292401 (PMC10581484; doi:10.1371/journal.pone.0292401)
Supplement: S2 File — (PDF) [file pone.0292401.s002.pdf]

Yeast strain genotypes

yBS1039 was used for all experiments in this study.

Strain name	Strain genotype	Notes
yWS677	S288C, BY4741 MATa his3Δ1 leu2Δ0 met15Δ0 ura3Δ0, sst2Δ0 far1Δ0 bar1Δ0 ste2Δ0 ste12Δ0 gpa1Δ0 ste3Δ0 mf(alpha)1Δ0 mf(alpha)2Δ0 mfa1Δ0 mfa2Δ0 gpr1Δ0 gpa2Δ0	Shaw, W. M., et al. (2019). "Engineering a Model Cell for Rational Tuning of GPCR Signaling." Cell 177(3): 782-796.e727.
yBS1010	yWS677, can1Δ0 fcy1Δ0	Simultaneous knock out of CAN1 and FCY1 genes, replaced with CRISPR addressable landing pads
yBS1027	yBS1010, URA3::LexO (6x)-pLEU2m-sfGFP-tTDH1 pCCW12 pFCY1-FCY1 inverted-CRISPR LP-URA3 first half inverted pPGK1-GPA1-Gαi1/2-tENO2 pRAD27-LexA-PRD-tENO1	Integration of refactored mating pathway genes, including FCY1+URA3 landing pad for selectable GPCR integration
yBS1039	yBS1027, URA3::LexO (6x)-pLEU2m-sfGFP-tTDH1 pCCW12-CB2-ymTagBFP2-tDIT1-Barcode URA3 pPGK1-GPA1-Gαi1/2-tENO2 pRAD27-LexA-PRD-tENO1	Integration of yeast codon-optimized human CB2-ymTagBFP2 with barcode sequence following tDIT1: CGATGAATTTTAAATGAAATTGT
