# Supplementary material for: Automation protocol for high-efficiency and high-quality genomic DNA extraction from Saccharomyces cerevisiae
Source: PLoS One. 2023 Oct 17;18(10):e0292401. doi: 10.1371/journal.pone.0292401 (PMC10581484; doi:10.1371/journal.pone.0292401)
Supplement: S1 Raw images — (PDF) [file pone.0292401.s005.pdf]

Raw gel images for figures in:
Automation Protocol for High-Efficiency and High-Quality Genomic DNA Extraction from Saccharomyces cerevisiae.

For Figs. 4, 6, 10, 11, and 12, the DNA ladder was: GeneRuler 1 kb Plus DNA Ladder (Thermo Scientific, cat. no. SM1333)

For Fig. 5, two DNA ladders were used: GeneRuler 1 kb Plus DNA Ladder and GeneRuler High Range DNA Ladder (Thermo Scientific, cat. no. SM1353)

All gel images were acquired using a FastGene FAST-Digi PRO gel documentation system (NIPPON Genetics, cat. no. GP-07LED)

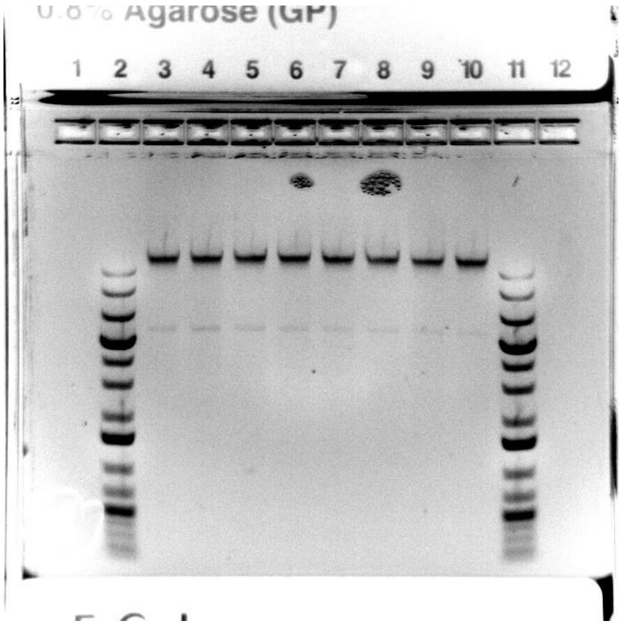

Figure 4A.

Lanes 3-10: samples A1-H1

Lanes 2 and 11: 1 kb Plus DNA ladder

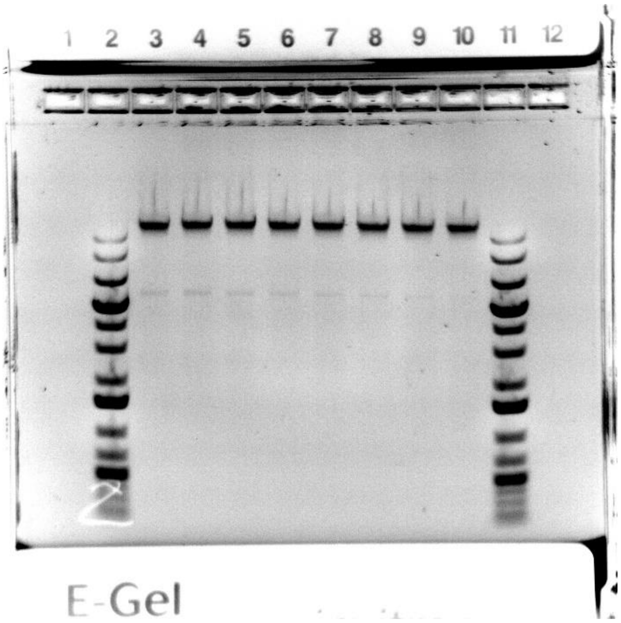

Figure 4A.

Lanes 3-10: samples A2-H2

Lanes 2 and 11: 1 kb Plus DNA ladder

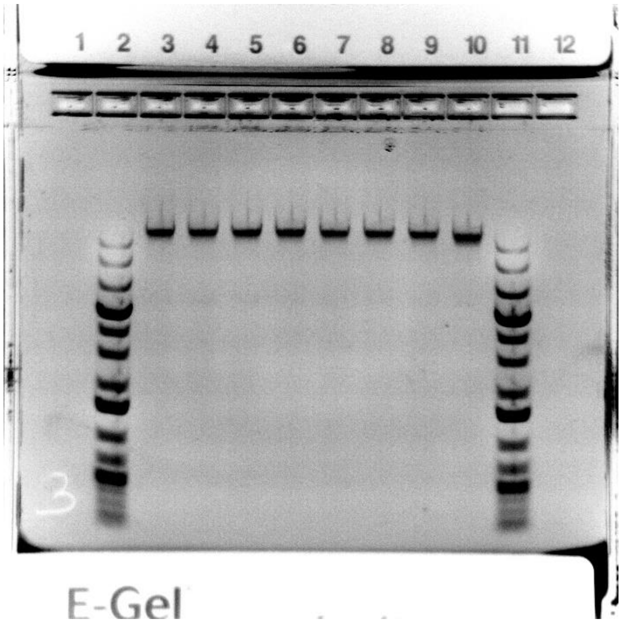

Figure 4A.

Lanes 3-10: samples A3-H3

Lanes 2 and 11: 1 kb Plus DNA ladder

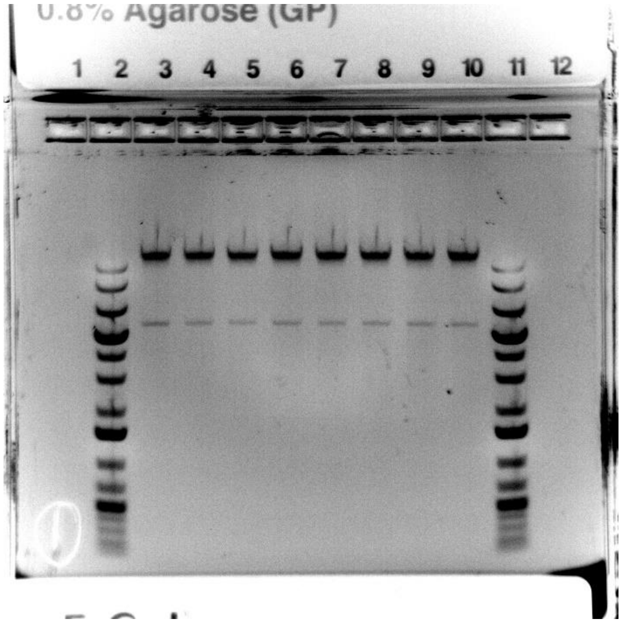

Figure 4B.

Lanes 3-10: samples A1-H1

Lanes 2 and 11: 1 kb Plus DNA ladder

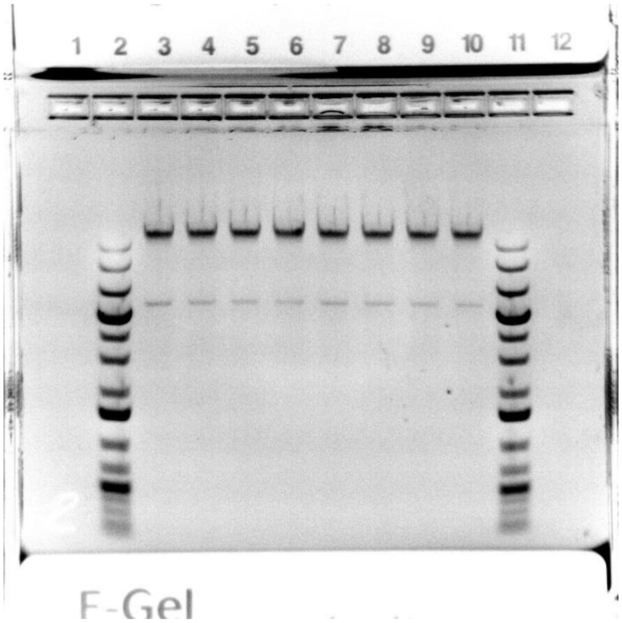

Figure 4B.

Lanes 3-10: samples A2-H2

Lanes 2 and 11: 1 kb Plus DNA ladder

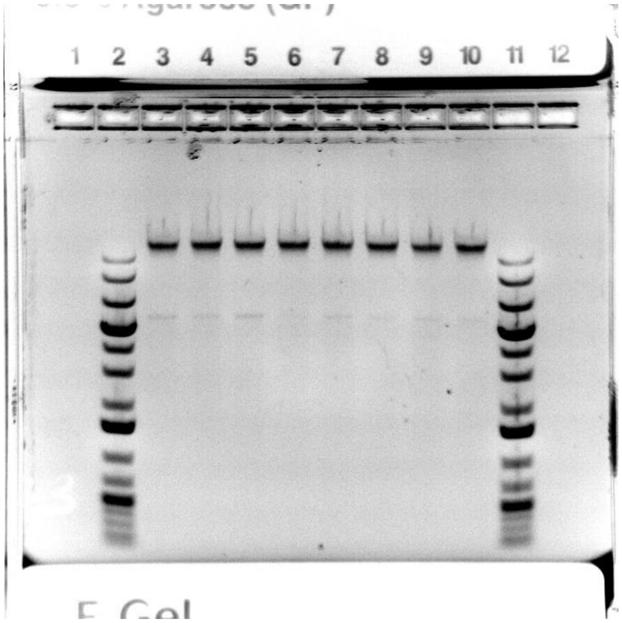

Figure 4B.

Lanes 3-10: samples A3-H3

Lanes 2 and 11: 1 kb Plus DNA ladder

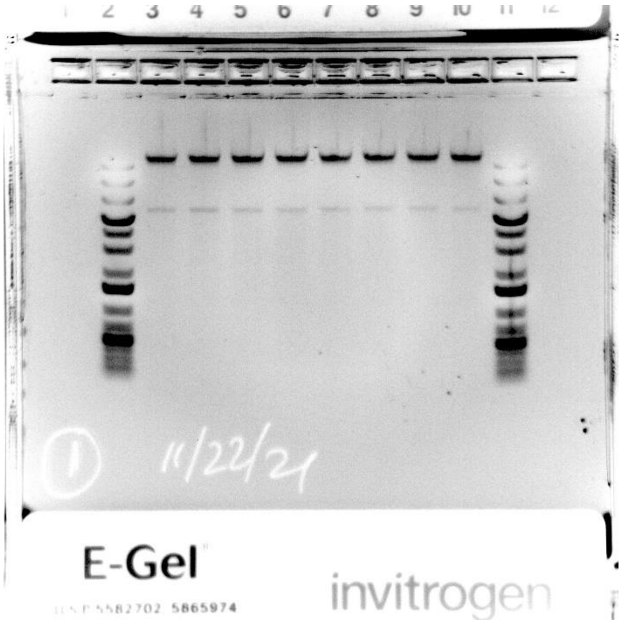

Figure 4C.

Lanes 3-10: samples A1-H1

Lanes 2 and 11: 1 kb Plus DNA ladder

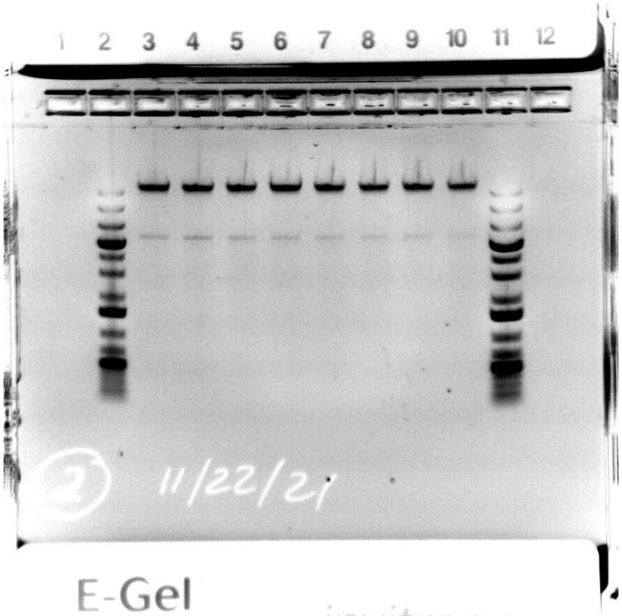

Figure 4C.

Lanes 3-10: samples A2-H2

Lanes 2 and 11: 1 kb Plus DNA ladder

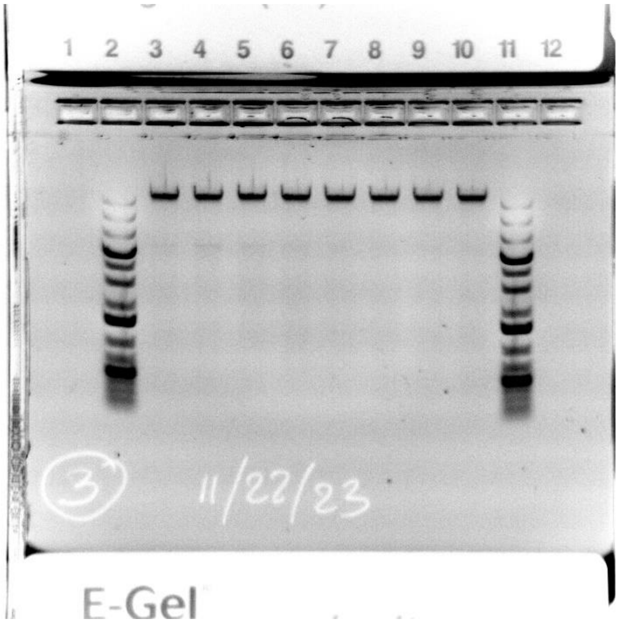

Figure 4C.

Lanes 3-10: samples A3-H3

Lanes 2 and 11: 1 kb Plus DNA ladder

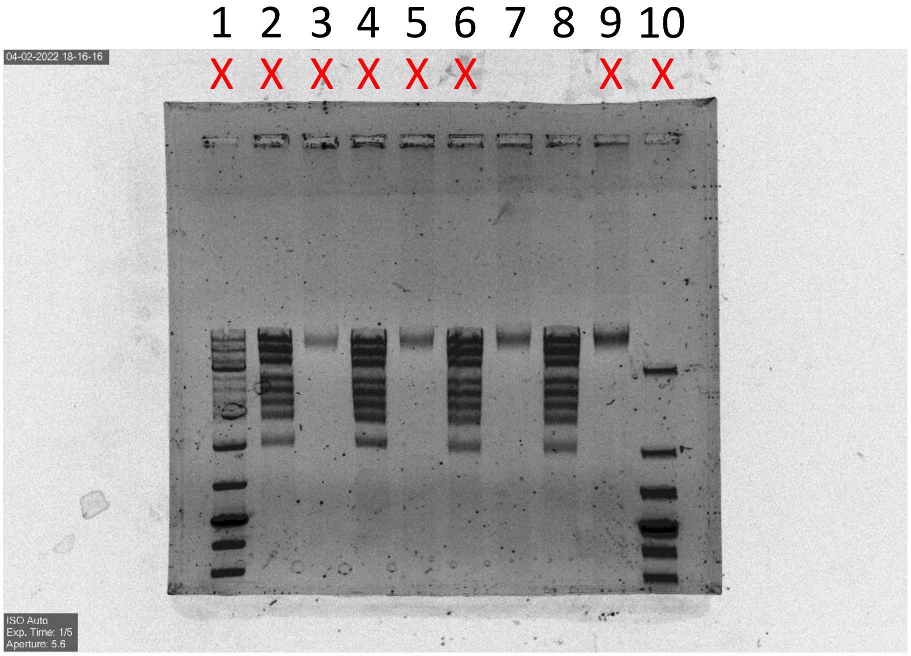

Figure 5.

Lane 1: Mixute of GeneRuler High Range DNA Ladder and GeneRuler 1 kb Plus DNA Ladder

Lanes 2, 4, 4, 8: GeneRuler High Range DNA Ladder

Lane 3: yeast gDNA prepared with our protocol, 2 µL sample load

Lane 5: yeast gDNA prepared with our protocol, 3 µL sample load

Lane 7: yeast gDNA prepared with our protocol, 4 µL sample load

Lane 9: yeast gDNA prepared with our protocol, 5 µL sample load

Lane 10: GeneRuler 1 kb Plus DNA Ladder

Only Lanes 7 and 8 are shown in manuscript Fig. 5

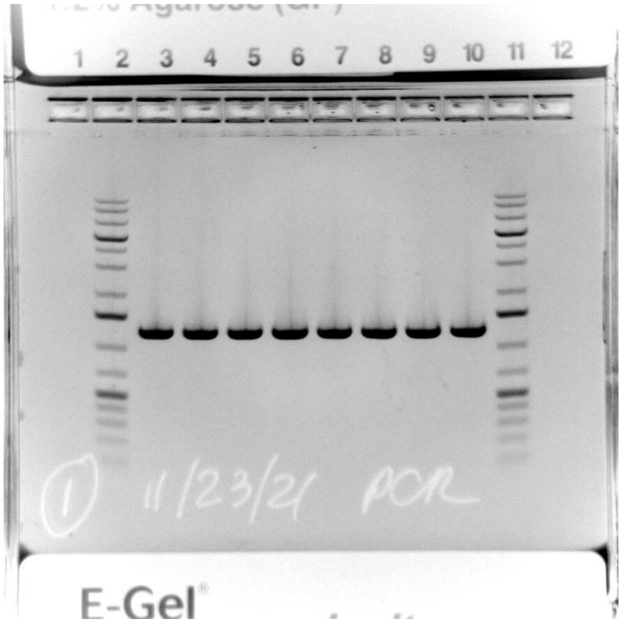

Figure 6A.

Lanes 3-10: samples A1-H1

Lanes 2 and 11: 1 kb Plus DNA ladder

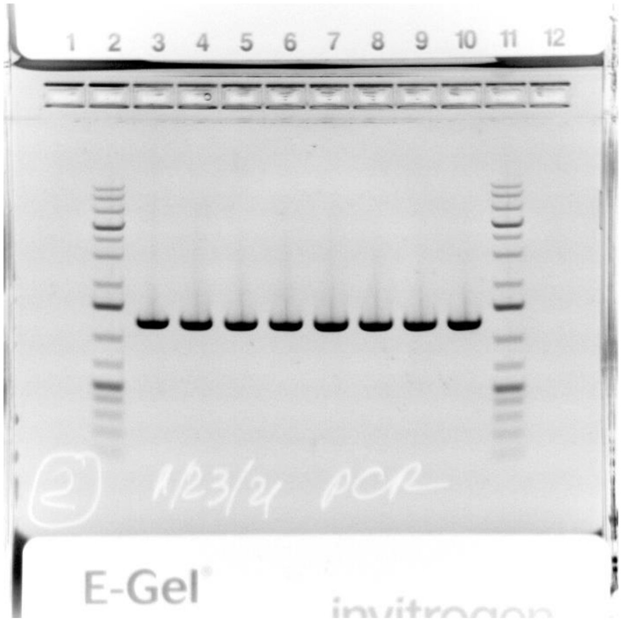

Figure 6A.

Lanes 3-10: samples A2-H2

Lanes 2 and 11: 1 kb Plus DNA ladder

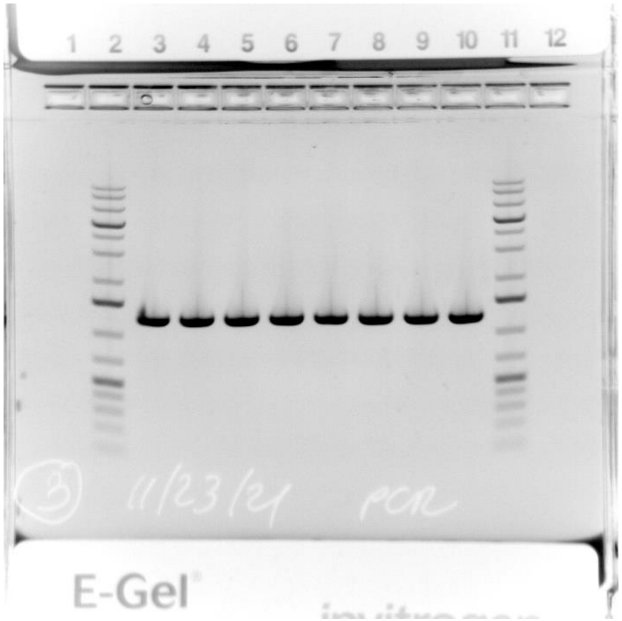

Figure 6A:

Lanes 3-10: samples A3-H3

Lanes 2 and 11: 1 kb Plus DNA ladder

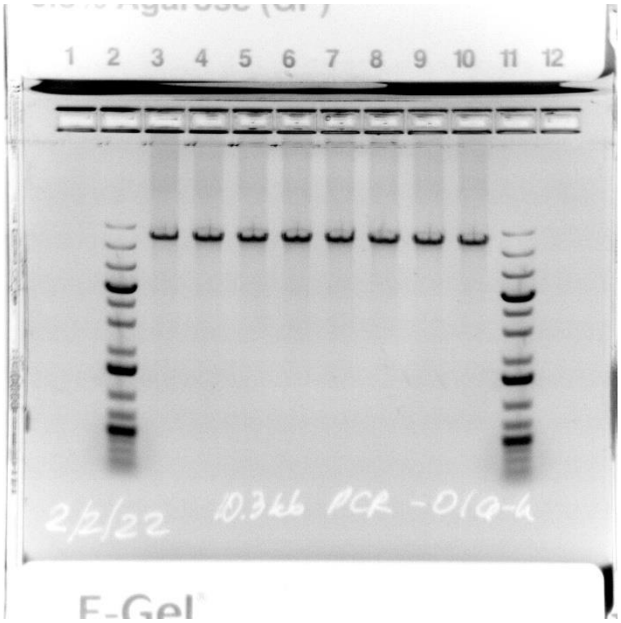

Figure 6B.

Lanes 3-10: samples A1-H1

Lanes 2 and 11: 1 kb Plus DNA ladder

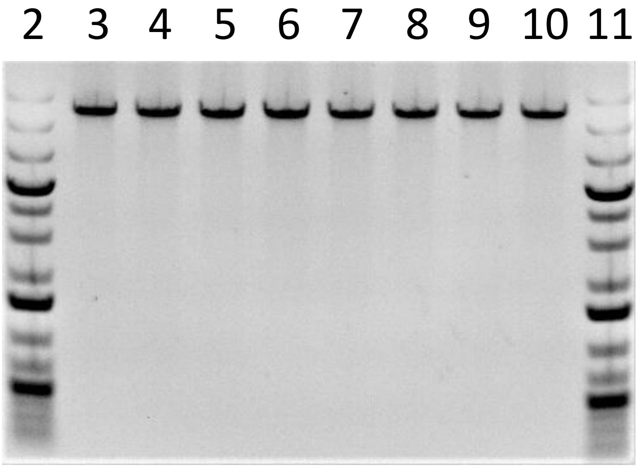

Figure 6B.
Full original gel image unavailable for samples A2-H2.
Image shown here has less cropping than in manuscript Fig. 6B.

Lanes 3-10: samples A2-H2
Lanes 2 and 11: 1 kb Plus DNA ladder

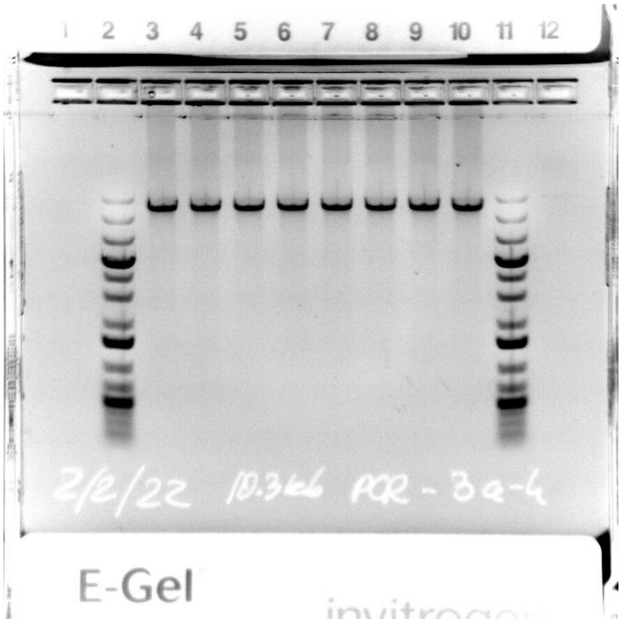

Figure 6B.

Lanes 3-10: samples A3-H3
Lanes 2 and 11: 1 kb Plus DNA ladder

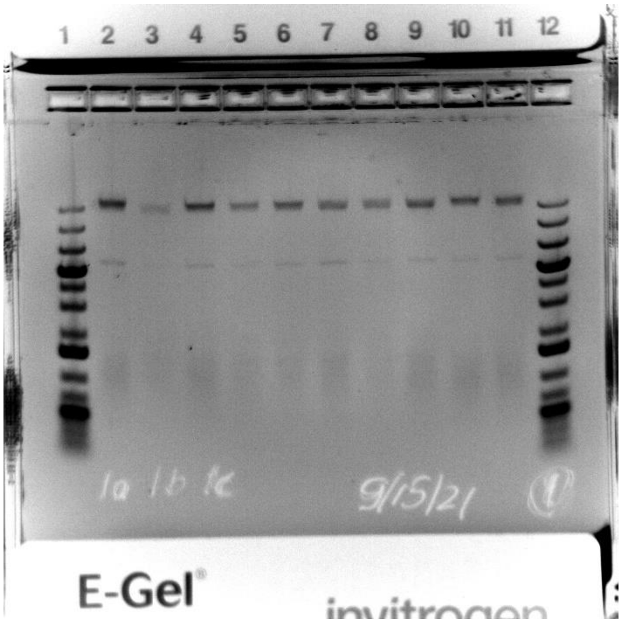

Figure 10A.

Lanes 2-9: samples A1-H1

Lanes 10-11: samples A2-B2

Lanes 1 and 12: 1 kb Plus DNA ladder

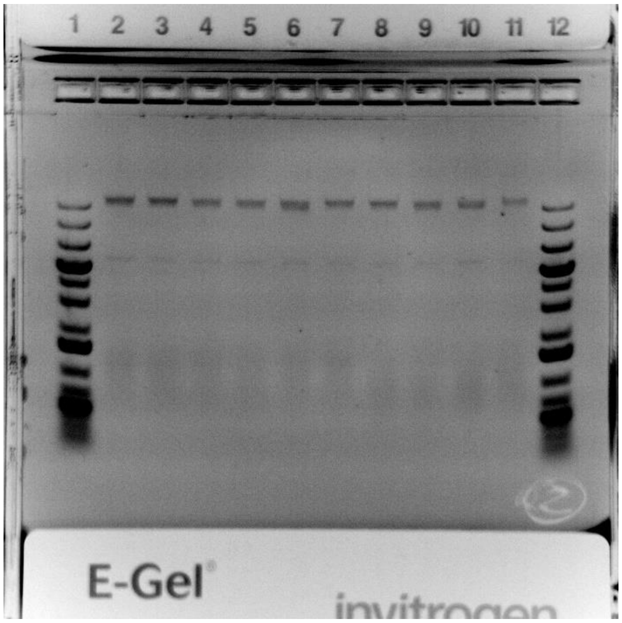

Figure 10A.

Lanes 2-7: samples C2-H2

Lanes 8-11: samples A3-D3

Lanes 1 and 12: 1 kb Plus DNA ladder

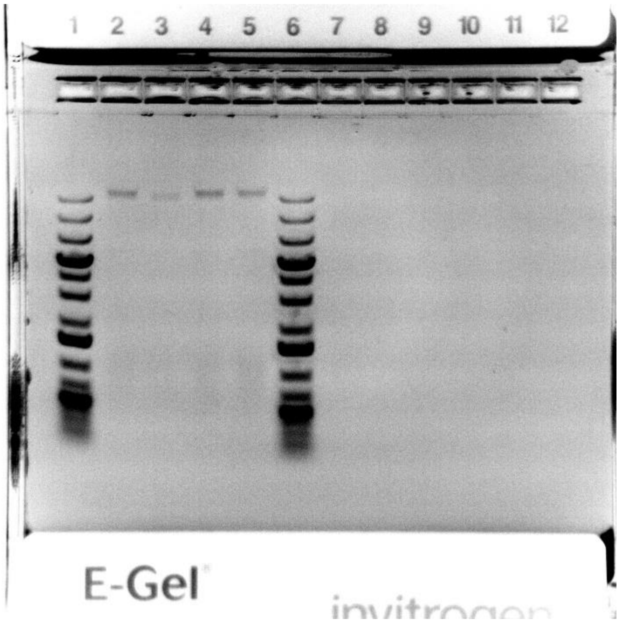

Figure 10A.

Lanes 2-5: samples E3-H3

Lanes 1 and 6: 1 kb Plus DNA ladder

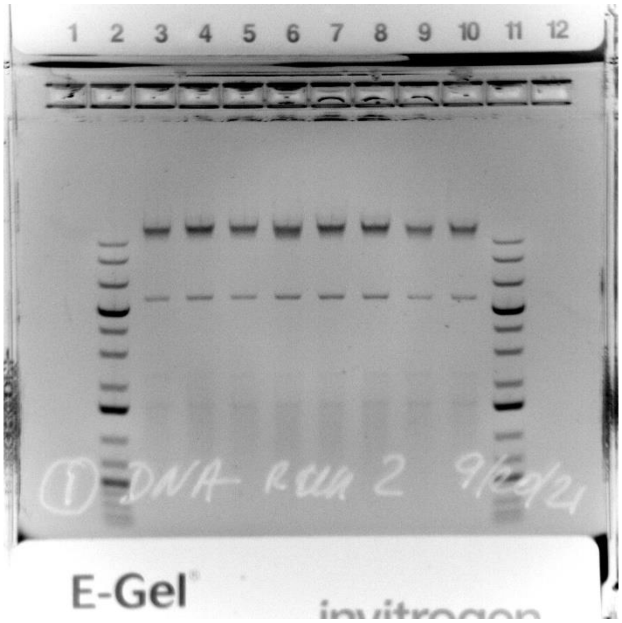

Figure 10B.

Lanes 3-10: samples A1-H1

Lanes 2 and 11: 1 kb Plus DNA ladder

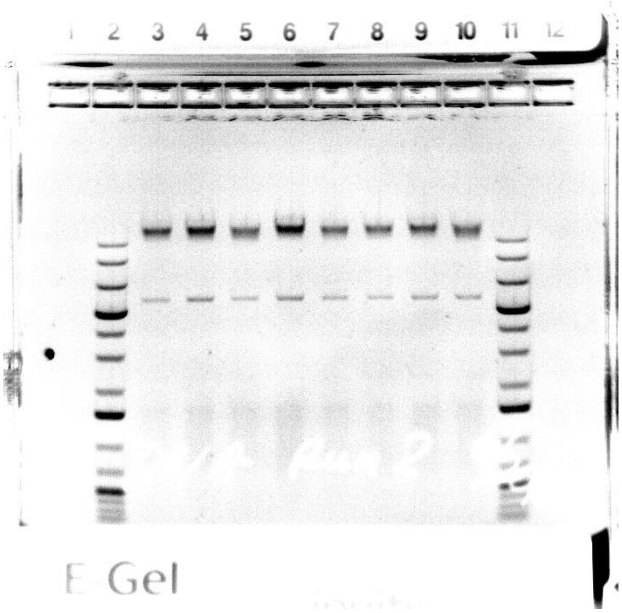

Figure 10B.

Lanes 3-10: samples A2-H2

Lanes 2 and 11: 1 kb Plus DNA ladder

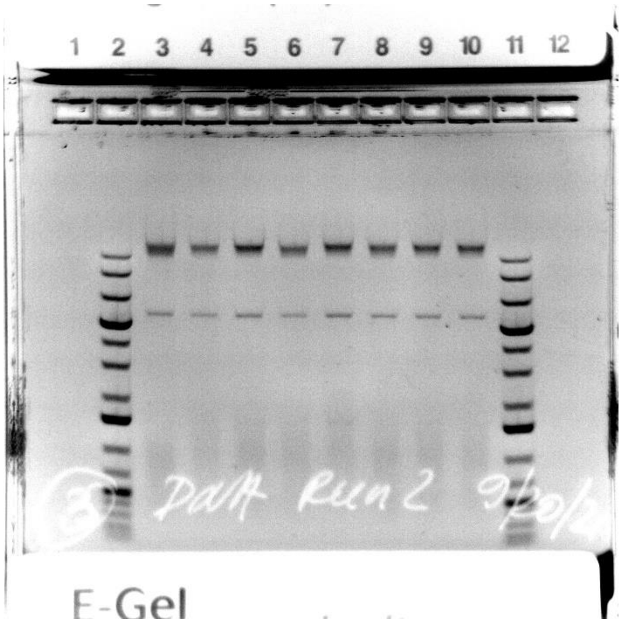

Figure 10B.

Lanes 3-10: samples A3-H3

Lanes 2 and 11: 1 kb Plus DNA ladder

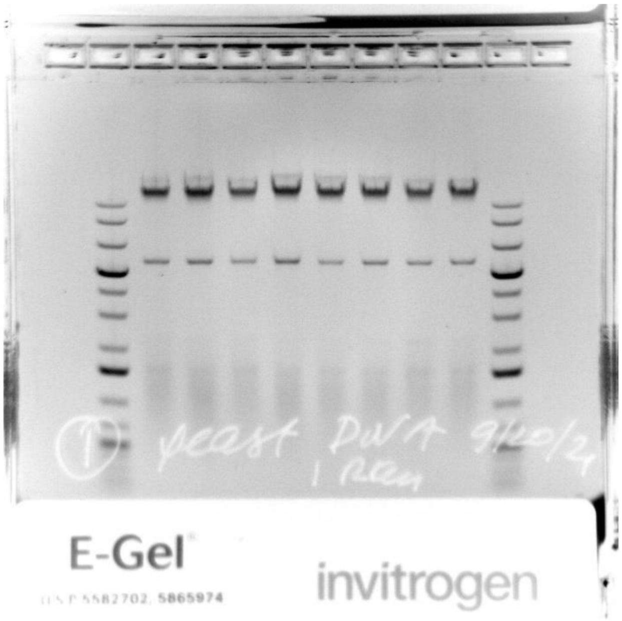

Figure 10C.

Lanes 3-10: samples A1-H1

Lanes 2 and 11: 1 kb Plus DNA ladder

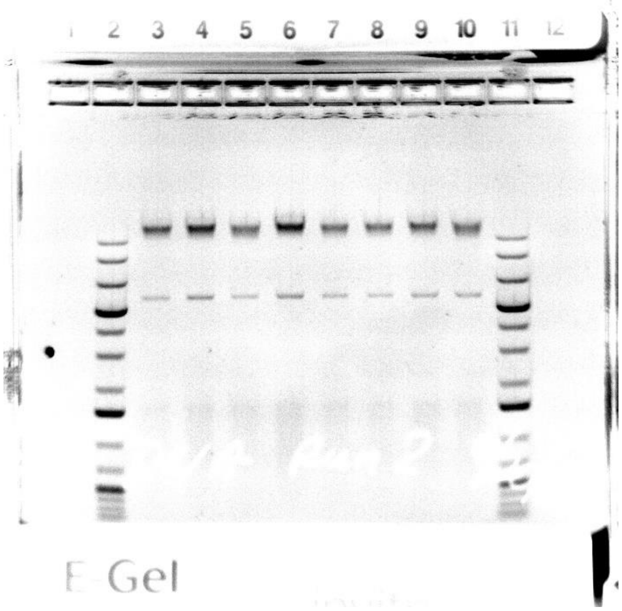

Figure 10B.

Lanes 3-10: samples A2-H2

Lanes 2 and 11: 1 kb Plus DNA ladder

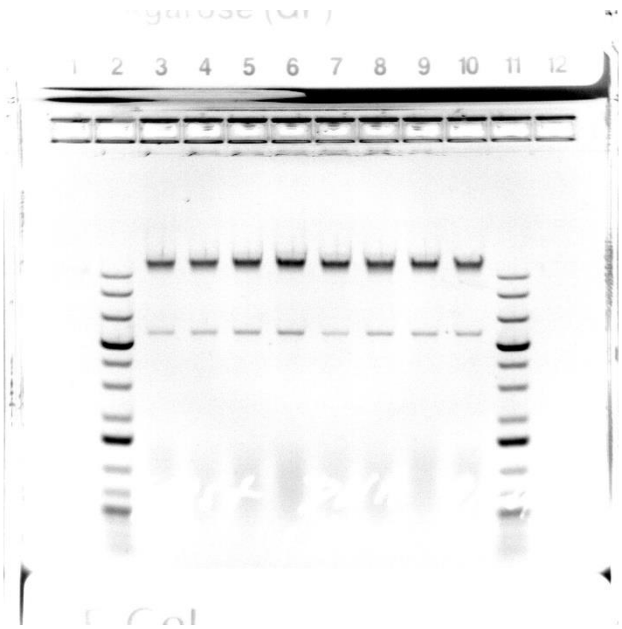

Figure 10C.

Lanes 3-10: samples A3-H3

Lanes 2 and 11: 1 kb Plus DNA ladder

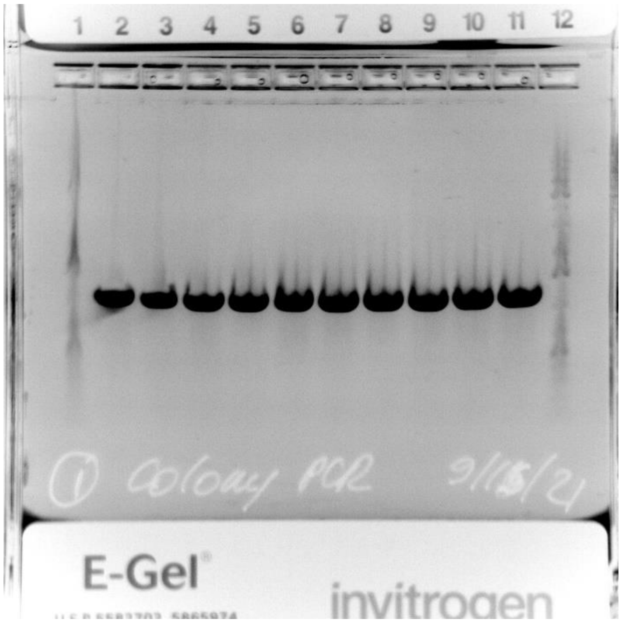

Figure 11A.

Lanes 2-9: samples A1-H1
Lanes 10-11: samples A2-B2
Lanes 1 and 12: 1 kb Plus DNA ladder

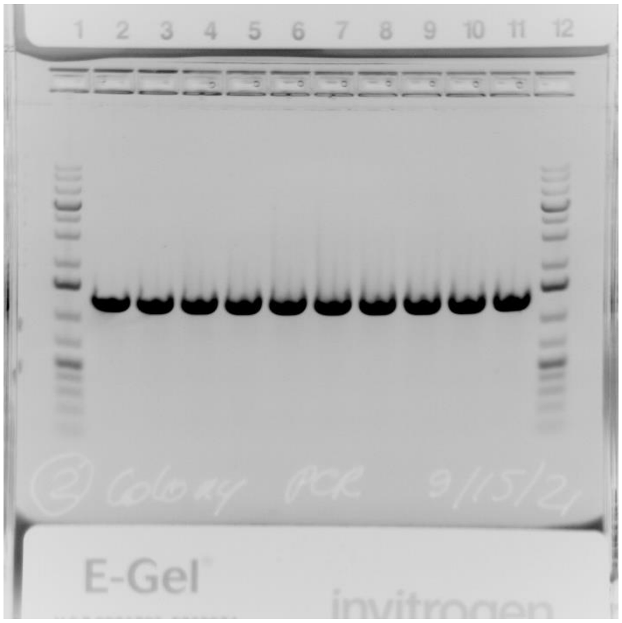

Figure 11A.

Lanes 2-7: samples C2-H2
Lanes 8-11: samples A3-D3
Lanes 1 and 12: 1 kb Plus DNA ladder

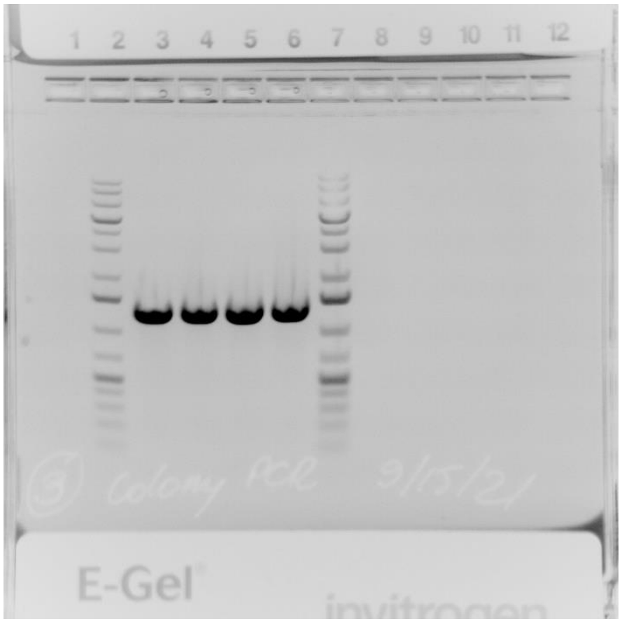

Figure 11A.

Lanes 3-6: samples E3-H3

Lanes 2 and 7: 1 kb Plus DNA ladder

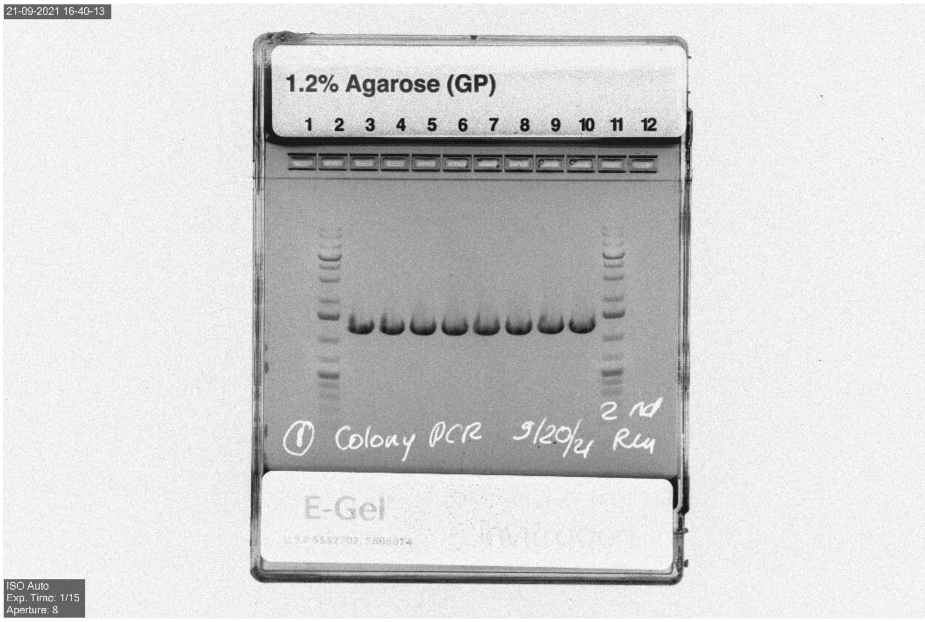

Figure 11B.

Lanes 3-10: samples A1-H1

Lanes 2 and 11: 1 kb Plus DNA ladder

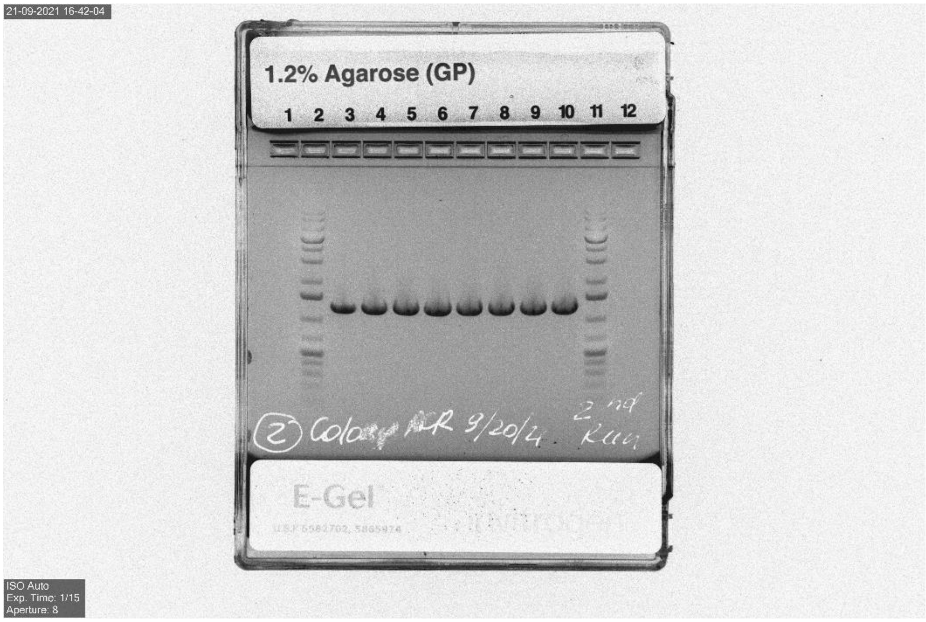

Figure 11B.

Lanes 3-10: samples A2-H2

Lanes 2 and 11: 1 kb Plus DNA ladder

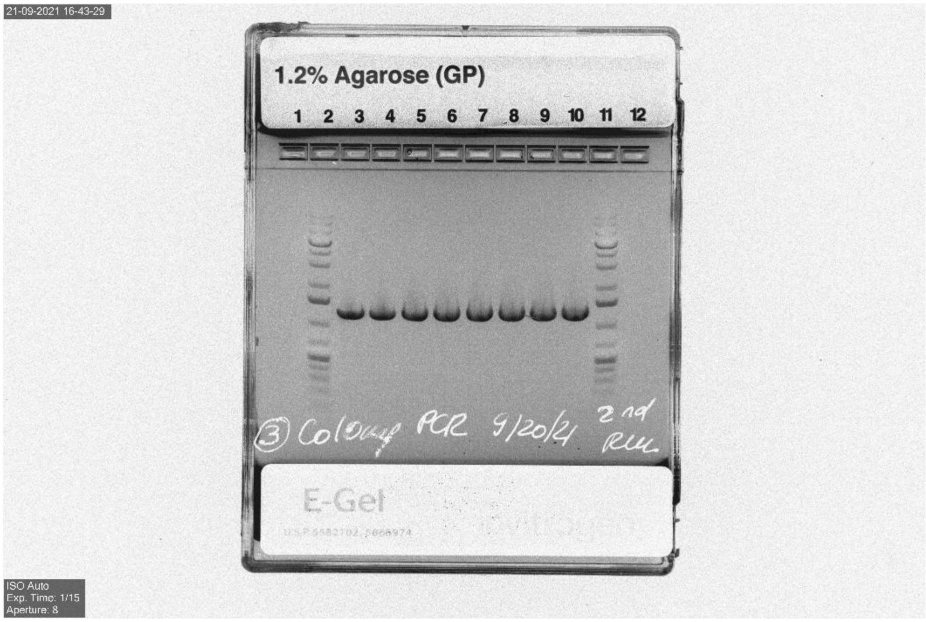

Figure 11B.

Lanes 3-10: samples A3-H3

Lanes 2 and 11: 1 kb Plus DNA ladder

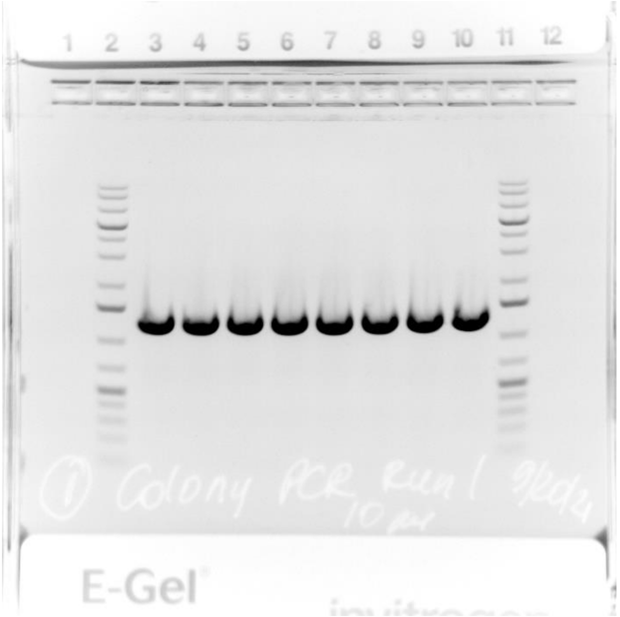

Figure 11C.

Lanes 3-10: samples A1-H1

Lanes 2 and 11: 1 kb Plus DNA ladder

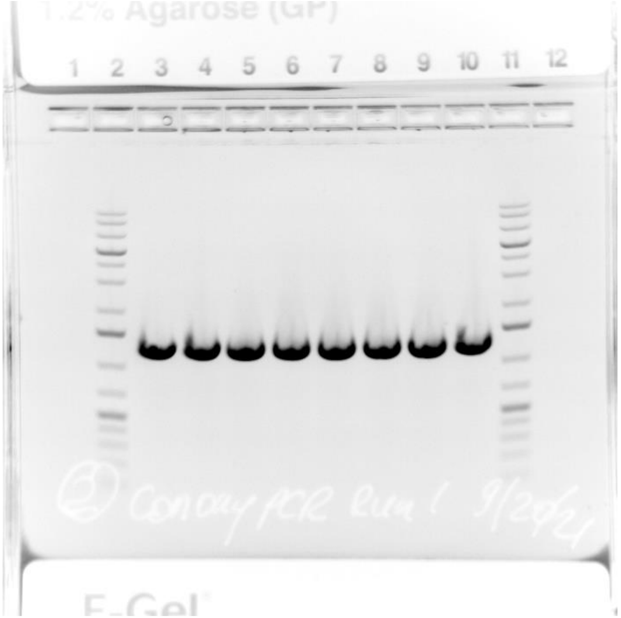

Figure 11C.

Lanes 3-10: samples A2-H2

Lanes 2 and 11: 1 kb Plus DNA ladder

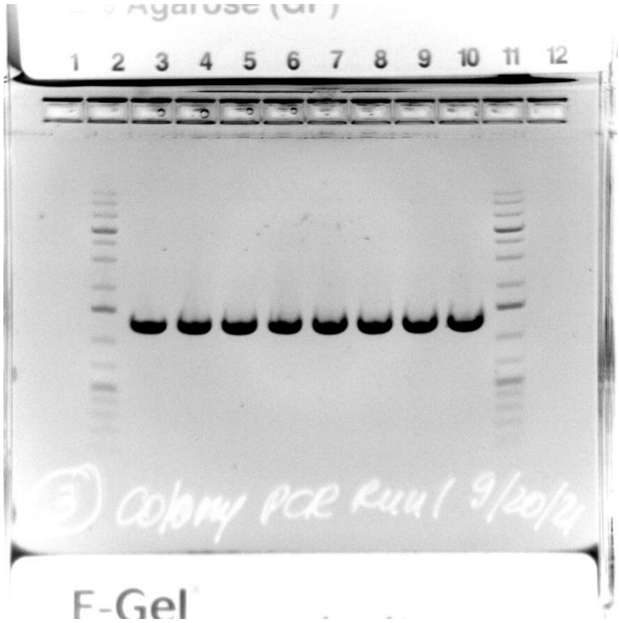

Figure 11C.

Lanes 3-10: samples A3-H3

Lanes 2 and 11: 1 kb Plus DNA ladder

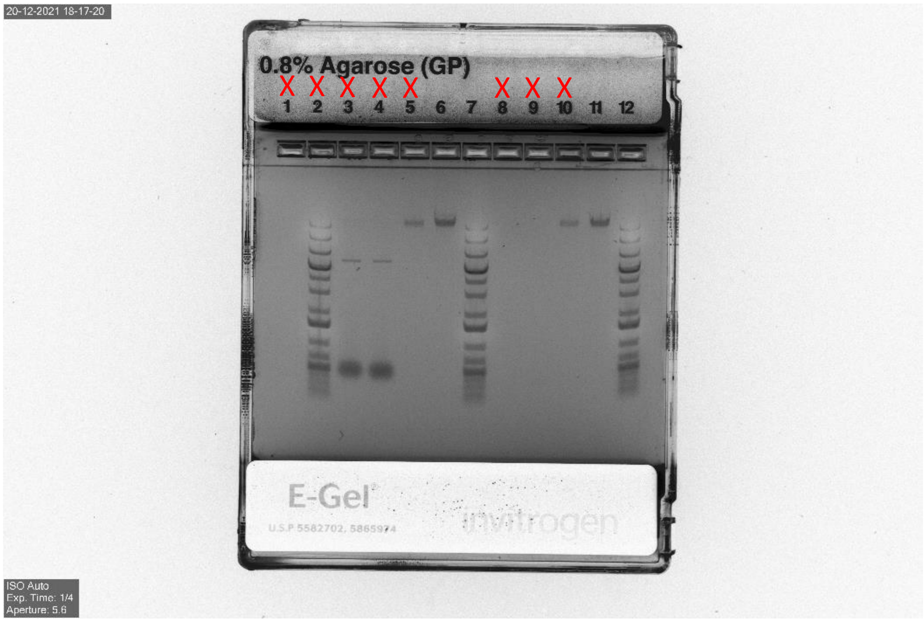

Figure 12A, left image; and Figure 12B, left image.

Lanes 2, 7, and 12: 1 kb Plus DNA ladder

Lane 3: yeast gDNA extracted using lithium-acetate-based protocol with centrifugation at 3000 g for 5 min., without RNase treatment

Lane 4: yeast gDNA extracted using lithium-acetate-based protocol with centrifugation at 15000 g for 3 min., without RNase treatment

Lane 5: yeast gDNA extracted using hybrid protocol using beginning of lithium-acetate-based protocol and continuing with our protocol (not included/described in manuscript), without RNase treatment

Lane 6: yeast gDNA extracted using our protocol, without extra RNase digestion

Lane 8: yeast gDNA extracted using lithium-acetate-based protocol with centrifugation at 3000 g for 5 min., with RNase treatment

Lane 9: yeast gDNA extracted using lithium-acetate-based protocol with centrifugation at 15000 g for 3 min., with RNase treatment

Lane 10: yeast gDNA extracted using hybrid protocol using beginning of lithium-acetate-based protocol and continuing with our protocol (not included/described in manuscript), with RNase treatment

Lane 11: yeast gDNA extracted using our protocol, with an extra RNase digestion step at the end of the extraction.

Only lanes 6, 7, 11, and 12 are included in the manuscript, Fig. 12.

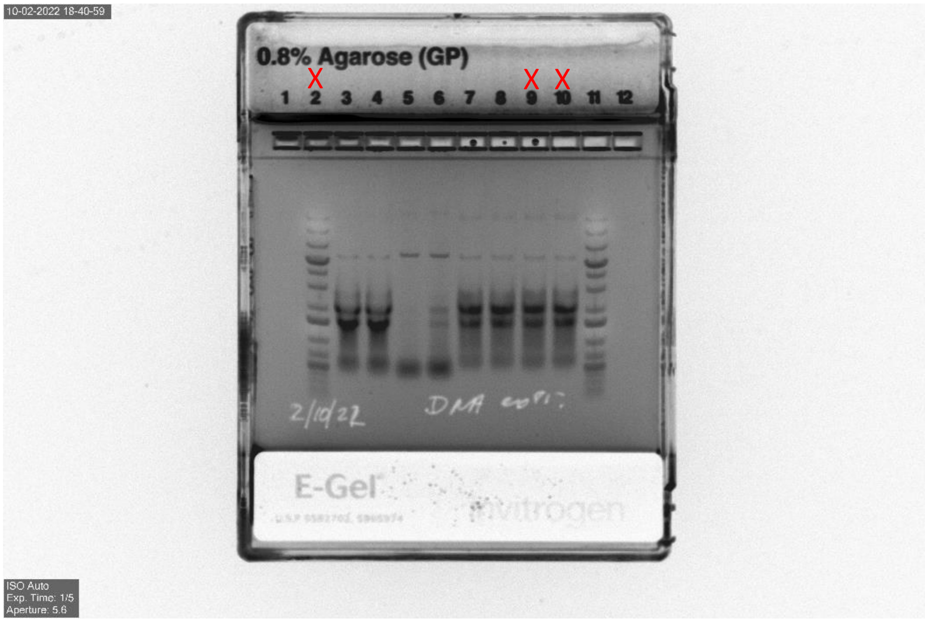

Figure 12A, middle image and right image.

Lanes 2 and 11: 1 kb Plus DNA ladder

Lanes 3-4: yeast gDNA extracted using fast, high-temperature SDS-based protocol, without RNase treatment

Lanes 5-6: yeast gDNA extracted using lithium-acetate-based protocol, without RNase treatment

Lanes 7-8: yeast gDNA extracted using commercial magnetic-bead-based kit, with incubation in T1 (SDS) buffer for 1 hour, without RNase treatment

Lanes 9-10: (not shown in manuscript Fig. 12A) yeast gDNA extracted using commercial magnetic-bead-based kit, with incubation in T1 (SDS) buffer for 2 hours, without RNase treatment

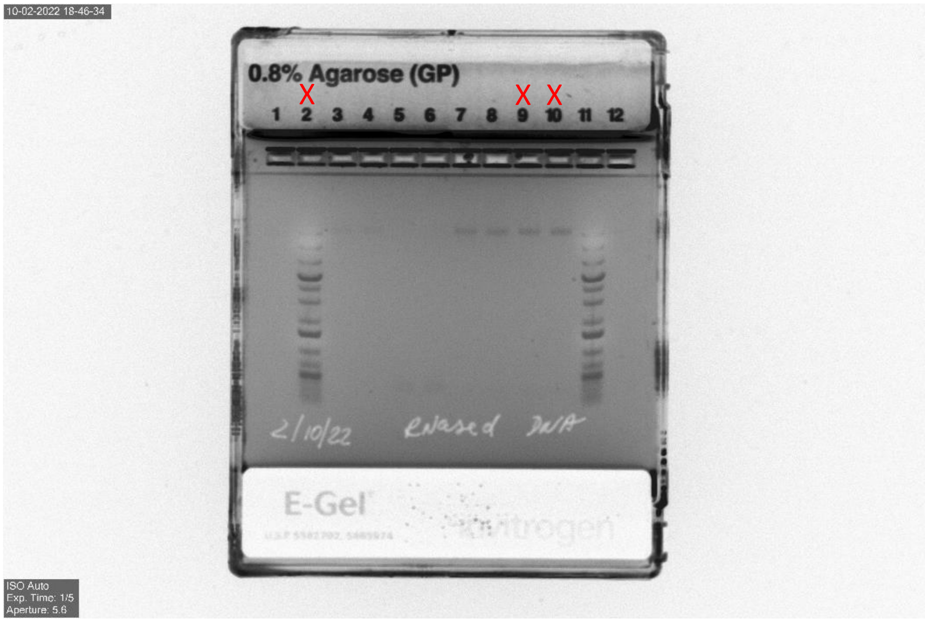

Figure 12B, middle image and right image.

Lanes 2 and 11: 1 kb Plus DNA ladder

Lanes 3-4: yeast gDNA extracted using fast, high-temperature SDS-based protocol, with RNase treatment

Lanes 5-6: yeast gDNA extracted using lithium-acetate-based protocol, with RNase treatment

Lanes 7-8: yeast gDNA extracted using commercial magnetic-bead-based kit, with incubation in T1 (SDS) buffer for 1 hour, with RNase treatment

Lanes 9-10: (not shown in manuscript Fig. 12B) yeast gDNA extracted using commercial magnetic-bead-based kit, with incubation in T1 (SDS) buffer for 2 hours, with RNase treatment
