# Supplementary material for: Automation protocol for high-efficiency and high-quality genomic DNA extraction from Saccharomyces cerevisiae
Source: PLoS One. 2023 Oct 17;18(10):e0292401. doi: 10.1371/journal.pone.0292401 (PMC10581484; doi:10.1371/journal.pone.0292401)
Supplement: Attachment — Submitted filename: Rebuttal Letter.docx [file pone.0292401.s006.docx]

We thank the reviewers for their feedback on the manuscript. Or point-by-point response to their comments are below (in blue).

Review Comments to the Author

Reviewer #1: This is a good, clear, professionally written paper that can be accepted almost in its current form. However, this study needs more discussion on the suitability of the extracted DNA for the NGS technique such as ONT. The lack of such debate is the primary flow of this paper. Authors should fill this gap. To do this, authors need to provide more measures of DNA purity, such as the standard Nanodrop measures (A260/280, A260/230, and Qubit\Nanodrop yield ratio (ideally should be 2:1)). Strictly speaking, the gel electrophoresis cannot test the quality of the DNA (line 90, p. 3). I do not see any issues linking their method with the cleanup procedure (upon necessity). It is unnecessary changing the protocol, but to suggest a few extra optional steps. It is unclear to the reader how pure the extracted DNA is, and this issue must be fixed as well (relevant suggestions on p. 2, lines 65 - 69 are insufficient for the reader).

There are different aspects to DNA quality that can be important for different down-stream applications. Gel electrophoresis can indicate whether the DNA is fragmented, for example, which is an important quality criterion for some applications. The absorbance ratios are also sometimes used for assessment of DNA purity, as noted by the reviewer. We have added information about the A260A280 and A260/A230 ratios which indicate the presence of protein continuation (as suspected by the reviewer). In addition, we added optional Proteinase K steps to the protocol, and indicated that they should be used for down-stream applications where protein contamination would be problematic.

Regarding the Qubit/Nanodrop ratio, ideally, both methods should give the same answer – since they are supposed to measure the same thing. However, Nanodrop measurements typically indicate a higher concentration than Qubit, particularly for low concentration samples. Though a ratio of Nanodrop:Qubit less than 2 is often considered to be ok. The Nanodrop:Qubit ratio for DNA extracted with our protocol was about 1.8. We have also added that information to the manuscript.

Minor - Table 1. (lines 148-150, 153). More references are necessary.

There are no citations for the commercial zymolyase-based kit or the commercial magnetic-bead-based kit tested in Table 1. We provide the full kit and vendor information in the Materials and Methods.

Reviewer #2: The protocol manuscript describes an automated protocol for extracting high quality DNA from the yeast S. cerevisae without using hazardous chemicals, boiling, or mechanical cell disruption. Although the mais objective of the paper is not clear in the Introduction, the manuscript is well written, the protocol’s steps are clearly described, and the results are robust. The conclusion could be greatly improved to discuss other similiar work that has already been published (for example 10.1128/jcm.40.6.2240-2243.2002 and 10.1111/j.1471-8286.2006.01428.x). In fact, this is the weakest point of the paper. What gap does exist in the literature that the present work fulfils, to justify its publication?

We appreciate the reviewer pointing out additional citations that might be relevant. The two papers recommended by the reviewer are for very different types of samples than the samples used in our protocol. The first was specific to medical samples and the second to mammalian tissue. Neither of those publications provides any assessment of the quality of the extracted DNA other than PCR assays. In addition, in the first recommended publication, the only description of the protocol is:

“For DNA extraction with the MagNA Pure LC, the MagNA Pure LC Total Nucleic Acid Isolation kit (Roche Diagnostics) was used… In the automated DNA isolation process, the samples were dissolved and simultaneously stabilized by incubation with buffer containing guanidinium thiocyanate and proteinase K, total nucleic acids were bound to the surface of glass magnetic particles, unbound substances were removed by several washing steps, and purified DNA was eluted with a low-salt buffer.”

Most of the details, including the composition of the reagents, are not given – presumably because that information is proprietary. So, if those two recommended citations are representative of protocol publications for DNA extraction, then I’d say that one clear gap that our manuscript fills is that we fully describe our protocol, in sufficient detail that other groups could implement it, and we extensively characterize the performance of our protocol – going well beyond the examples recommended by the reviewer or any other similar publications that we’ve been able to find.

Additional gaps are identified in the manuscript’s introduction: Given the widespread use of Saccharomyces cerevisiae in the field of engineering biology and the increased use of laboratory automation, we think it is important to provide a well-documented and tested automation protocol specific to that organism.

It should also be mentioned that the protocols used for comparison are either intrinsically bad or were poorly executed because all yielded bad results. This should be explained or thoroughly reviewed.

We disagree with the reviewer’s opinion on the protocols used for comparison. When we asked other people in the yeast engineering biology community for recommended gDNA extraction protocols, they recommended the protocols that we used for comparison in this manuscript. And although those protocols are good enough for applications that require something simple like a short PCR product from a clonal population, they were not good enough for our applications that require high efficiency and low RNA contamination. Furthermore, although we got lower DNA yields from the comparison methods than what was reported in the original papers on those methods, that discrepancy could be due to differences in the culture conditions (which are not typically described in any detail).

To address the reviewer’s request here, we have added a short comparison of the results we obtained with the comparison methods and the expected results based on yields reported in the papers describing those methods.
